# Small intestine metastasis from cervical cancer with acute abdomen: A case report

**DOI:** 10.3892/ol.2014.2659

**Published:** 2014-11-03

**Authors:** HUI QIU, LIMEI YUAN, YANGWEN OU, YAN ZHU, CONGHUA XIE, GONG ZHANG

**Affiliations:** Department of Oncology, Zhongnan Hospital of Wuhan University, Hubei Cancer Clinical Study Center and Hubei Key Laboratory of Tumor Biological Behavior, Wuhan, Hubei 430071, P.R. China

**Keywords:** cervical cancer, small intestine, acute abdomen, metastasis

## Abstract

Cervical cancer metastasis to the small intestine is a rare occurrence that is easily misdiagnosed as a small bowel obstruction. The present study reports the case of a 46-year-old cervical cancer patient with metastasis to the small intestine, which presented as an acute abdomen due to intestinal obstruction. Enteroscopy revealed no primary intestinal tumors. The patient underwent exploratory laparotomy and resection of the tumor of the small intestine. Pathology revealed the mass to be squamous cell carcinoma, limited to the outer muscular layer and serosa. This case demonstrates that small intestine seeding must be considered in the differential diagnosis of acute abdomen in patients with cervical cancer.

## Introduction

Cervical cancer has been established as the second most common cancer among females worldwide (1,2). Cervical cancer is a serious heath problem and the majority of cases occur in developing countries (3), as no effective screening procedures are available (4). Recently, with the improvement of cervical cancer screening, the worldwide incidence and mortality of cervical cancer has decreased (5). However, the incidence of cervical cancer in young individuals worldwide has markedly increased and exhibits a poor prognosis (6,7). Patients with localized disease may be cured after definitive cancer therapy, and previous studies have indicated that surgery or radiation therapy provide an equivalent outcome (8). Patients that present with regional or distant disease are at a greater risk of mortality (9). Common metastases of cervical cancer include local extension and lymph node and pulmonary metastasis. However, metastasis to the small intestine is rare and, to the best of our knowledge, it has not been reported in the literature in the previous several decades. Small intestine metastasis from primary tumors located elsewhere in the body easily results in a missed or incorrect diagnosis, as the metastasis is frequently regarded as acute abdomen, with the main symptom of abdominal pain (10,11). The present study presents and discusses a case of cervical cancer with symptomatic small intestine metastasis.

## Case Report

**Figure 1:**
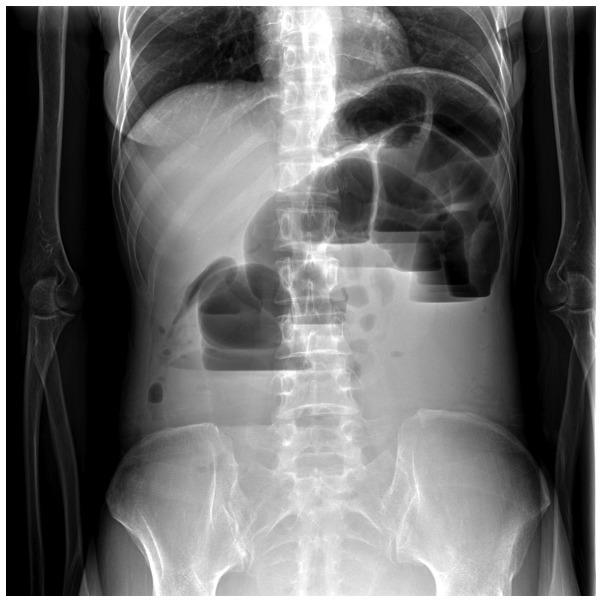
Abdominal X-ray revealing multiple liquid-gas surfaces.

A 46-year-old female was admitted to the gastroenterology department of the Zhongnan Hospital of Wuhan University (Wuhan, China) due to acute abdominal pain. Abdominal examination revealed mild tenderness without rebound tenderness, with decreased peristalsis, detected by auscultation. In the initial laboratory tests, the serum carcinoembryonic antigen level was 5.46 ng/ml (normal range, 0–7.2 ng/ml); ferritin, 370.62 ng/ml (normal range, 12–150 ng/ml); squamous cell carcinoma antigen, 9.9 ng/ml (normal range, 0–1.5 ng/ml) and sodium ion, 133.3 mmol/l (normal range, 135–145 mmol/l). The results of a routine analysis of the blood [white blood cell count, 4.0×10^9^/l (normal range, 4.0–10.0×10^9^/l); red blood cell count, 3.6×10^12^/l (normal range, 3.5–5.5×10^12^/l); hemoglobin, 114 g/l (normal range, 110–150 g/l); platelet count, 125×10^9^/l, (normal range, 100–300×10^9^/l); neutrophil percentage, 62% (normal range, 50–70%); lymphocyte percentage, 25% (normal range, 20–40%); monocyte percentage, 5.2% (normal range, 3–8%); acidophilic cell percentage, 0.7% (normal range, 0.5–5%); and basophilic cell percentage, 0.1% (normal rane, 0–1%)], urinalysis, a liver function test [Aspertate aminotransferase (AST), 28 U/l (normal range, 0–46 U/l); Alanine aminotransferase (ALT), 35 U/l (normal range, 0–46 U/l); AST/ALT ratio, 0.81 (normal range, 0.2–2.0); total bilirubin, 17.7 μmol/l (normal range, 0–25 μmol/l); direct bilirubin, 6.8 μmol/l (normal range, 0–7 μmol/l); indirect bilirubin, 10.9 μmol/l (normal range, 1.5–18 μmol/l); total protein, 65 g/l (normal range, 60–80 g/l); albumin, 38 g/l (35–55 g/l); globulin, 24 g/l (normal range, 20–30 g/l); albumin/globulin ratio, 1.58 (normal range, 1.5–2.5); glutamine, 51 U/l (normal range, 5–55 U/l); alkaline phosphatase, 132 U/l (normal range, 35–134 U/l); and total bile acids, 6.4 μmol/l (normal range, 0–15 μmol/l)] and chest films all normal. On observation, the abdominal X-ray revealed multiple liquid-gas surfaces, suggesting intestinal obstruction (Fig. 1). In addition, the patient had been diagnosed with stage IIB cervical cancer in 2010 and treated by definitive chemoradiotherapy consisting of whole pelvic external beam radiotherapy of 50 Gy in 25 fractions, with center shielding and concomitant high-dose rate intracavitary brachytherapy with 192-iridium remote after loading system for 42 Gy to the intersection of the vaginal vault. The concurrent chemotherapy regimen was cisplatin, 40 mg/m^2^/week. The patient was subsequently diagnosed with lower intestinal obstruction and cervical cancer following chemoradiotherapy.

**Figure 2:**
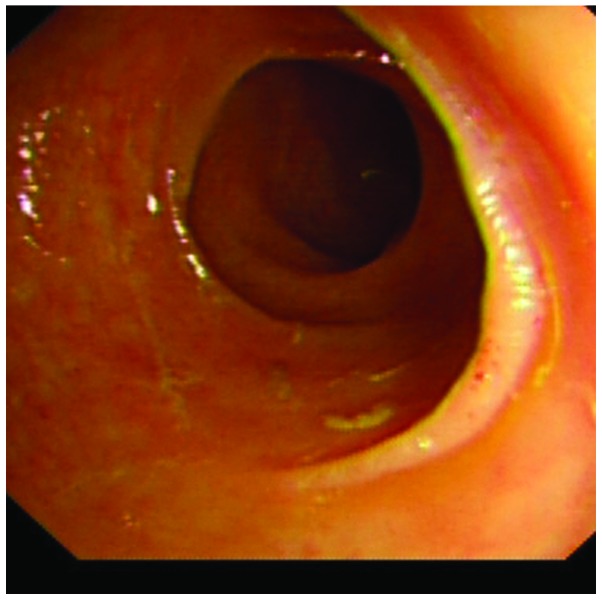
Colonoscopy confirming that the intestinal mucosa was smooth without ulcers or lumps.

**Figure 3:**
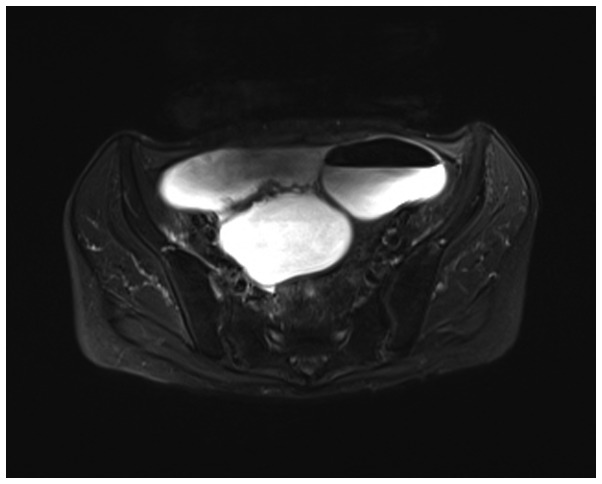
Magnetic resonance imaging of pelvic cavity revealing the lower intestinal obstruction.

**Figure 4:**
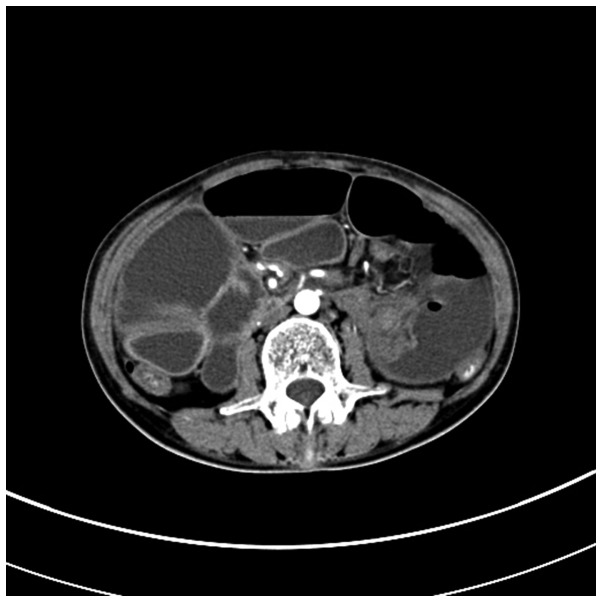
Abdominal computed tomography scan revealing lower intestinal obstruction.

The mechanical bowel obstruction was proposed to have been caused by an advanced complication following pelvic radiotherapy, or a small intestine primary or metastatic tumor. The patient was provided with anti-inflammatory treatment, gastric tube drainage, acid suppression, fluid infusion and nutritional support. Concomitantly, colonoscopy revealed that the intestinal mucosa was smooth without any ulcer or lump (Fig. 2). Magnetic resonance imaging of the pelvic cavity showed the change in cervical cancer following chemoradiotherapy, pelvic effusion and lower intestinal obstruction (Fig. 3). A computed tomography (CT) scan of the abdomen revealed cervical cancer following chemoradiotherapy and lower intestinal obstruction (Fig. 4). As the effect of conservative treatment was not satisfactory, with gradually worsening abdominal pain, the patient was transferred to the department of general surgery. Under general anesthesia, laparotomy was performed, which revealed the significant expansion of the small intestine, little ascites, multiple pelvic nodules, wide small mesenteric lymph node enlargement and a mass that was approximately 30.0 cm in size, which originated from the ileocecal junction and caused the complete occlusion of the intestine.

**Figure 5:**
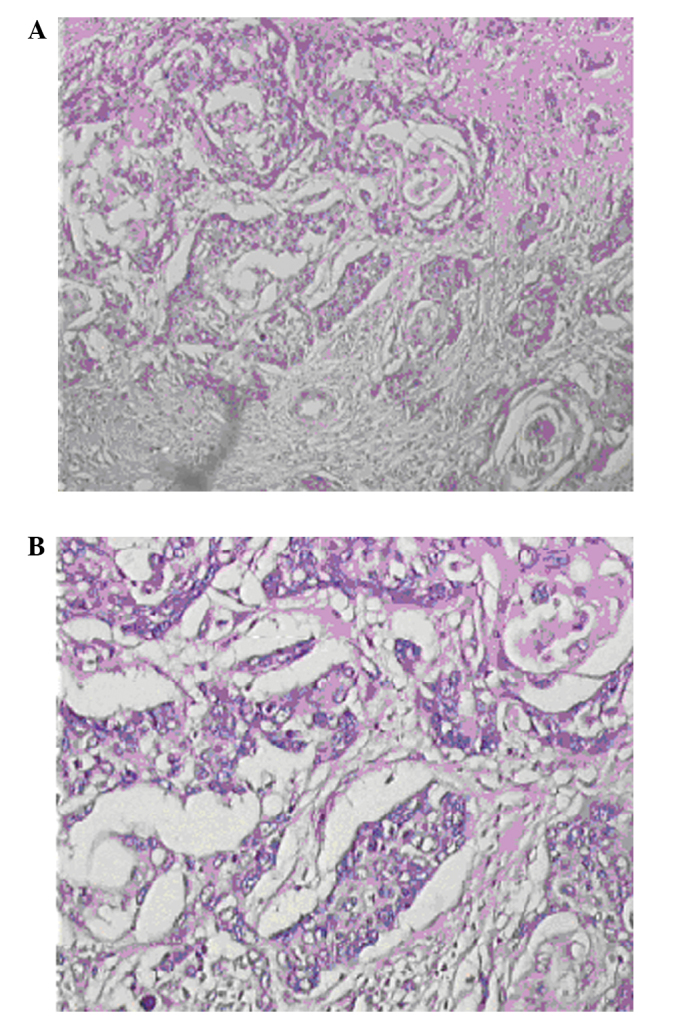
Microscopic examination of the intestinal tumor demonstrating typical histological findings of squamous cell carcinoma (staining with hematoxylin and eosin). (A) Magnification, ×100 and (B) magnification, ×400.

The resection of the small intestinal tumors and ileostomy was immediately performed. A pathological diagnosis of squamous carcinoma was determined, and cancer tissue was limited to the outer muscular layer and serosa (Fig. 5). A final diagnosis of cervical cancer with small intestine metastases was determined. Postoperatively, the ileus symptoms improved and the general condition of the patient also improved. The patient was treated with four cycles of a docetaxel-cisplatin combination chemotherapy regimen (day 1, 75 mg/m^2^ docetaxel; days 1–3, 25 mg/m^2^ cisplatin, every 21 days). One month following chemotherapy, the patient returned to the hospital for regular follow-up appointments, which were subsequently attended every three months for two years.

Written, informed consent was obtained from the patient for the publication of the present study and the related images.

## Discussion

Cervical cancer is the second most common cancer in women, being second only to breast cancer (12). The traditional treatment is radical surgery, with radiotherapy and chemotherapy predominantly used for the treatment of advanced or recurrent patients. In 2001, the National Comprehensive Cancer Network recommended cisplatin-based concurrent chemoradiotherapy as the standard treatment for advanced and high-risk early cervical cancer. Over the past decade, treatment with concurrent chemoradiotherapy has evidently prolonged the survival of patients with cervical carcinoma (13–15). However, with the improvement of survival, the patients are also at increased risk of recurrence and metastases.

When cervical carcinoma metastasizes, it usually does so via local or lymphatic dissemination. Hematogenous dissemination occurs less frequently, can spread to almost all organs and usually affects the lungs initially, followed by the bones and paraaortic, intraperitoneal and supraclavicular lymph nodes (16). Cervical cancer metastasis to the small intestine is rare, and easily misdiagnosed as it is frequently regarded as acute abdomen. According to the main diagnosis standard of metastatic small intestinal tumors (17), it must first be clear where the primary tumors are located. Secondly, the patient presents with serious clinical complications, including perforation, obstruction or hemorrhage. Thirdly, the tumor must be histopathologically confirmed and, finally, it must arise neither from direct infiltration nor abdominal metastasis. In the present case, combined with the histopathological characteristics of the patient and the history of cervical squamous cell carcinoma, the patient was finally diagnosed with squamous cell carcinoma with metastasis to the small intestine.

It is generally considered that small intestine metastasis does not easily occur, due to the intensive lymphoid tissue in intestinal wall, which can produce immunoglobulin to enhance immunity (18). It has been reported that small intestine metastasis occurs in approximately 4–10.6% of cancer cases (19). The stomach, colon and ovary are common primary tumor sites (20). Small intestinal tumors are likely to present with intussusception and intestinal obstruction with bowel stricture or expansion, and thus, surgeons must investigate the possibility of bumps close to the lesions. As metastasis from cervical cancer is considered unlikely, and the lesions are often considered to be advanced symptoms or side-effects of chemotherapy, the metastases are difficult to identify. When patients present with abdominal symptoms, including abdominal pain, nausea, vomiting, anemia and weight loss, or CT scans depict short segmental bowel-wall thickening or a polypoid mass in the small intestine in combination with regional lymphadenopathy, perforation, or intussusception, the gastrointestinal tract of the patient must be meticulously examined to enable early detection and treatment. At the present time, comparison between positron emission tomography-CT, abdominal contrast-enhanced CT and endoscopy is the most effective method for determining digestive tract metastasis (21). In the current study, the patient’s condition deteriorated following chemotherapy, and this was hypothesized to be due to small intestine metastasis.

The present case indicates that if cervical cancer patients present with small intestine obstruction, small intestine metastasis must be considered in the differential diagnosis of the condition as acute abdomen.
